# Characterization of InSb Nanoparticles Synthesized Using Inert Gas Condensation

**DOI:** 10.1186/s11671-015-0966-4

**Published:** 2015-06-10

**Authors:** Sneha G Pandya, Martin E Kordesch

**Affiliations:** 156 Clippinger Laboratories, Department of Physics and Astronomy, Ohio University, Athens, OH 45701 USA

**Keywords:** InSb nanoparticles, Inert gas condensation, XRD, Williamson-Hall analysis, HRTEM, FTIR

## Abstract

Nanoparticles (NPs) of indium antimonide (InSb) were synthesized using a vapor phase synthesis technique known as inert gas condensation (IGC). NPs were directly deposited, at room temperature and under high vacuum, on glass cover slides, TEM grids and (111) p-type silicon wafers. TEM studies showed a bimodal distribution in the size of the NPs with average particle size of 13.70 nm and 33.20 nm. The Raman spectra of InSb NPs exhibited a peak centered at 184.27 cm^−1^, which corresponds to the longitudinal optical (LO) modes of phonon vibration in InSb. A 1:1 In-to-Sb composition ratio was confirmed by energy dispersive X-ray (EDX). X-ray diffractometer (XRD) and high-resolution transmission electron microscopy (HRTEM) studies revealed polycrystalline behavior of these NPs with lattice spacing around 0.37 and 0.23 nm corresponding to the growth directions of (111) and (220), respectively. The average crystallite size of the NPs obtained using XRD peak broadening results and the Debye-Scherrer formula was 25.62 nm, and the value of strain in NPs was found to be 0.0015. NP’s band gap obtained using spectroscopy and Fourier transform infrared (FTIR) spectroscopy was around 0.43–0.52 eV at 300 K, which is a blue shift of 0.26–0.35 eV. The effects of increased particle density resulting into aggregation of NPs are also discussed in this paper.

## Background

Indium antimonide (InSb) is a well-known III-IV semiconductor with one of the smallest band gaps (~0.17 eV at 300 K) and the highest room-temperature electron mobility (~78,000 cm^2^/(Vs)^2^). InSb has a very small effective mass for electrons and thus has a large Bohr radius of ~65 nm. All these properties make InSb a good candidate for infrared detectors, magnetic sensors, cooling devices, thermoelectric power generation, high-speed field-effect transistors, and low-power device applications [1–4]. Low-dimensional InSb structures show good quantum confinement and various studies have been published on synthesis and characterization of InSb thin films and nano-wires [5–11]. But on the other hand, InSb nanoparticles (NPs) have been rarely studied. The synthesis of low dimensional InSb structures has been challenging in general and problems like non-uniformity in size of NPs and aggregation of NPs have persisted. Here, we present a comparatively straightforward method for synthesis of InSb NPs with reasonable control over NP aggregation.

We have synthesized InSb NPs using a vapor phase technique known as inert gas condensation (IGC). The synthesis process will be described in detail in this paper. NPs were directly deposited on substrates at room temperature. The NPs were characterized using techniques like X-ray diffractometer (XRD), transmission electron microscopy (TEM)—regular and high-resolution mode, energy dispersive X-ray (EDX) spectroscopy, scanning optical Raman (SOR) spectroscopy, and Fourier transform infrared (FTIR) spectroscopy.

## Methods

**Fig. 1:**
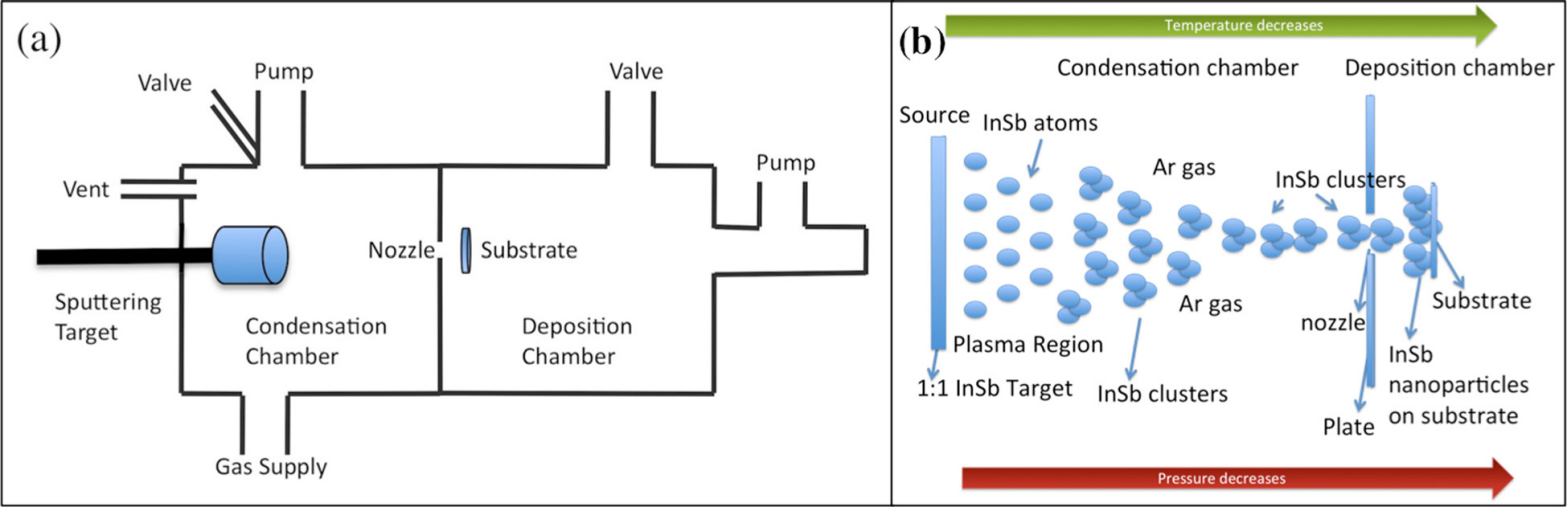
**a** Schematic of the inert gas condensation instrument used in our laboratory. **b** Diagram explaining the formation of the nanoparticles during the inert gas condensation process

InSb NPs were synthesized using a vapor phase technique known as IGC. It is a bottom-up process in which individual atoms, ions, and molecules condense together to form NPs. The schematic of the instrument used in our laboratory can be seen in Fig. 1a. It comprises of two chambers, condensation chamber and deposition chamber. Radio frequency magnetron sputtering is used as the source to produce individual atoms, ions, and molecules of InSb in the condensation chamber. The adjacent chamber, known as the deposition chamber, is separated from the condensation chamber by a small nozzle, which is either a hole of 2 mm diameter or a 20-mm long and 1-mm wide slit. The condensation chamber is under constant supply of Ar gas, and the deposition chamber is under constant vacuum pumping. This causes a high pressure difference in between these two chambers. Due to the pressure difference, the individual atoms, ions, and molecules of InSb produced during the sputtering process move away from the plasma region, where they collide with Ar ions and lose their energy and momentum and condense to form clusters of InSb. The pressure difference between these chambers results in the formation of a beam of InSb clusters, and their subsequent extraction from the condensation chamber in form of NPs. These NPs are then directly deposited onto a substrate that is placed exactly in front of the nozzle, at a distance of about 1–2 cm, in deposition chamber.

A diagram explaining the formation of NPs in the condensation chamber can be seen in Fig. 1b. A 99.99 % pure 1:1 InSb target, 38.1 mm in diameter and 3.175 mm in thickness, was sputtered in presence of 99.99 % pure Ar gas. The base pressure of the system was maintained at 10^−7^ Torr. The parameters used for synthesis were as follows: power =30–50 W, condensation chamber pressure =1 T, and deposition time =30–90 min. The ratio of pressure in the condensation chamber to that of deposition chamber was maintained at 1000. The aggregation length, defined as the distance between sputtering source and the nozzle, was 10 cm, and the distance between the substrate and the nozzle was 1 cm. Various substrates like glass cover slides, TEM grids, and (111) p-type silicon wafers were used to facilitate various types of NP characterization.

Structural characterization of NPs was carried out using a Rigaku MiniFlex-II X-Ray Diffractometer operated at 30 kV and 15 mA (with CuKα radiation at 0.154 nm) and a JEOL 1010 TEM operated at 100 kV and JEM 2100F high-resolution transmission electron microscopy (HRTEM) system. The composition of InSb NPs was obtained using a Noran Instruments EDX assembled with a S-2460N Hitachi thermionic emission SEM and operated at 25 kV accelerating voltage. The Raman spectrum was obtained using WITec R-SNOM-300s. A CW 532-nm wavelength YAG laser with adjustable power was used to excite the NPs. And the band gap of the NPs was obtained using a Perkin Elmer Spectrum Spotlight 300 FTIR microscope.

## Results and discussions

**Fig. 2:**
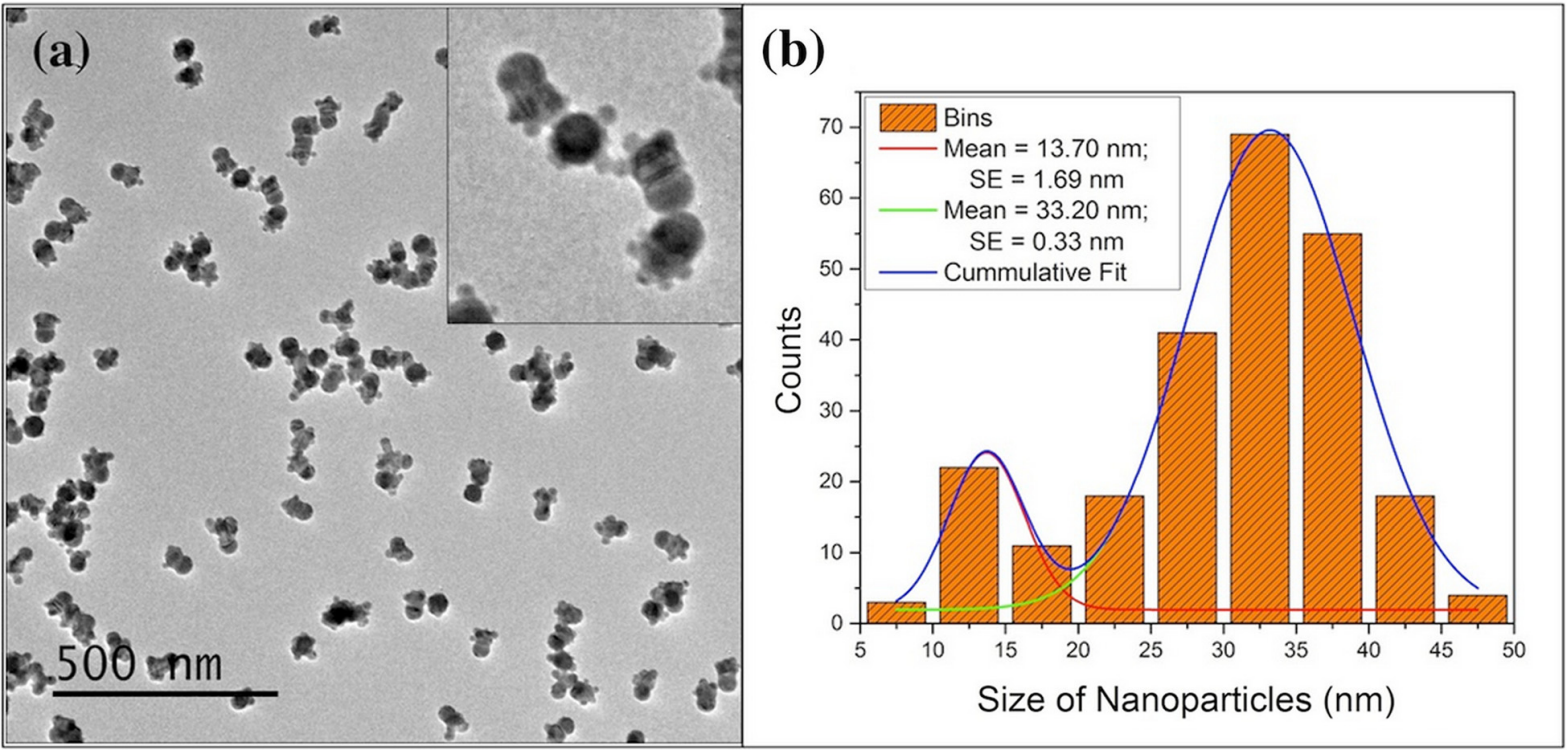
**a** TEM image for InSb NPs directly deposited on TEM grids by IGC process. *Inset* shows the magnified image for these NPs. **b** Size distribution histogram for TEM image seen in **a** showing a bimodal distribution with average NP size of 13.70 and 33.20 nm

InSb NPs synthesized using the abovementioned parameters are shown in the TEM image in Fig. 2a along with their size distribution histogram in Fig. 2b. The histogram shows a bimodal size distribution with the mean particle size of 13.70 and 33.20 nm. Individual NPs are formed of two to three smaller NPs of around 7–10 nm. The variation in the size of the NPs can be interpreted as follows: the condensation zone in IGC involves nucleation and growth processes. Depending on the synthesis parameters, either nucleation dominates growth or vice versa. The size of the NPs reduces if nucleation process dominates and enlarges if the growth process dominates. Experimentally, the nucleation and growth of the particles are mainly determined by factors like the cooling rate and the density in the nucleation and the growth region. Homogeneous nucleation is specifically affected by the cooling rate. After nucleation, the NP growth process follows continuously. The growth process is divided into two categories for an IGC process: coalescence and coagulation. Coalescence is a process in which the sputtered entities sinter and diffuse within the particles when entities come in contact, and coagulation refers to the process where two or more nuclei are join by processes such a Brownian motion. Coalescence occurs provided that the temperature is sufficiently high and results into spherical particles whereas coagulation occurs at lower synthesis temperatures and results into loose agglomerates with open structures. At intermediate temperature, partially sintered and non-spherical NPs are formed. So looking at our results, we can say that the synthesis temperature and the cooling rate cause the variation in the NP size along with their partial sintering.

**Fig. 3:**
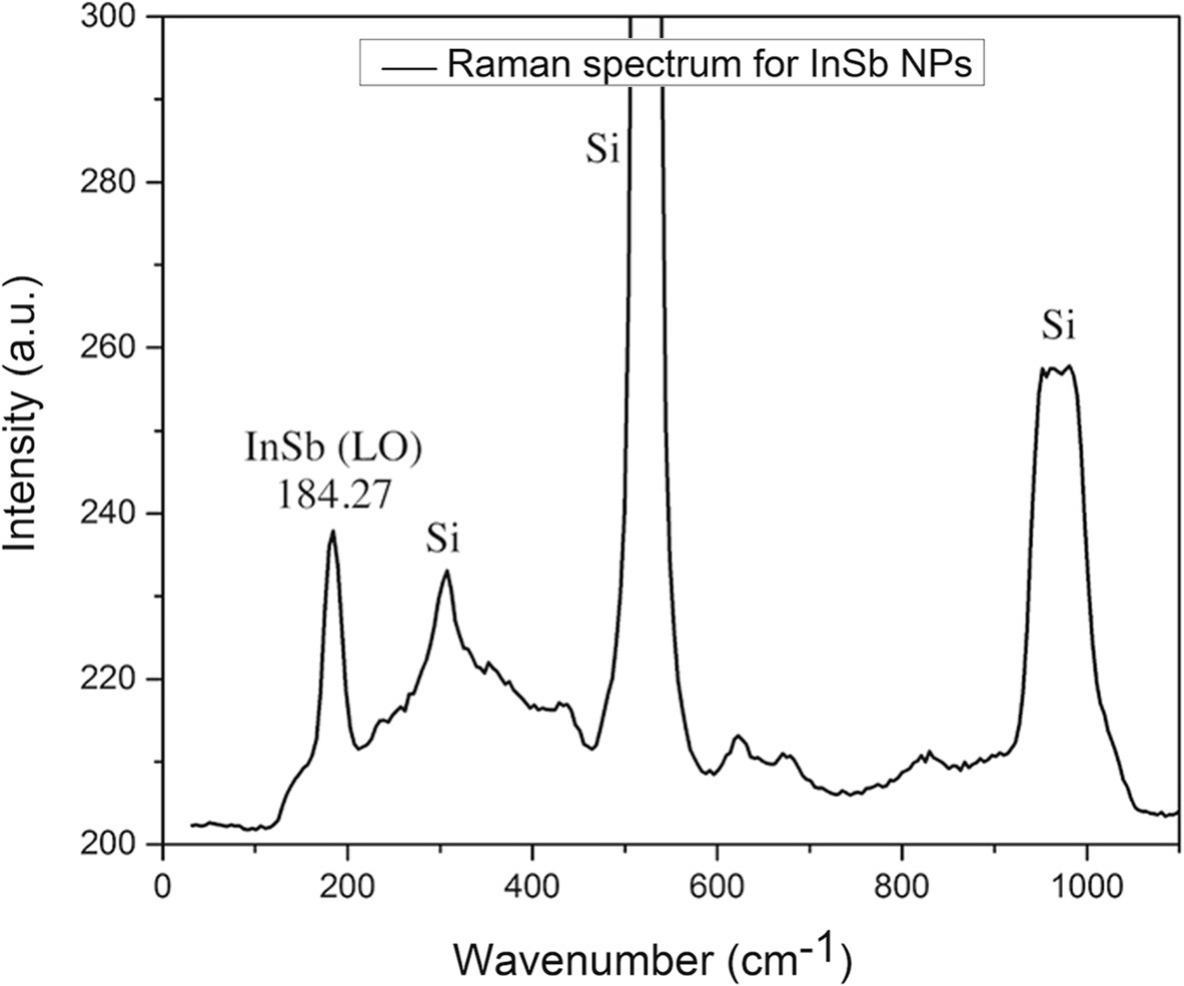
Raman spectra of InSb NPs deposited on a Si wafer. The peak at 184.27 cm^−1^ corresponds to the LO mode of phonon vibration in InSb. The remaining peaks come from the Si substrate

It can be seen from the TEM image that there is hardly any agglomeration of the NPs, and the NPs are uniformly distributed. The confinement effects seen in the optical study can be associated to this size. In order to change the size and particle density (PD) of the NPs, the synthesis parameters can be varied. For this particular paper, we have varied the power and deposition time to change the PD of the NP deposition. Power even affects the sizes of the NPs to a certain extent. The composition of the as-synthesized NPs was obtained using Raman spectroscopy studies at room temperature. Figure 3 shows the Raman spectrum for InSb NPs deposited on Si substrate. The peak at around 184.27 cm^−1^ corresponds to the longitudinal optical (LO) mode of phonon vibrations in InSb, thus confirming the formation of pure InSb NPs. To further confirm this, composition EDX studies were carried out. Table 1 shows the EDX data for the InSb NPs synthesized using IGC. We can see that the In-to-Sb ratio is approximately 1:1. The presence of carbon is the result of the instrument’s incapability to deal with smaller atomic number elements. Silicon observed here is due to the substrate.

**Table 1:** Composition in InSb NPs using EDX

Element line	*K*-ratio	Weight %	Weight % error	Atom %	Atom % error	Formula
C K	0.01	5.97	±0.45	14.78	±1.11	C
Si K	0.85	76.34	±0.18	80.77	±0.19	Si
In L	0.07	9.06	±0.37	2.35	±0.10	In
In M	0.00	–	–	–	–	
Sb L	0.07	8.63	±0.46	2.11	±0.11	Sb
Sb M	0.00	–	–	–	–	
Total		100.00		100.00		

EDX data for the InSb NPs synthesized using IGC process. The NPs were directly deposited on (111) p-type Si wafer. In-to-Sb ratio is almost 1:1 here

**Fig. 4:**
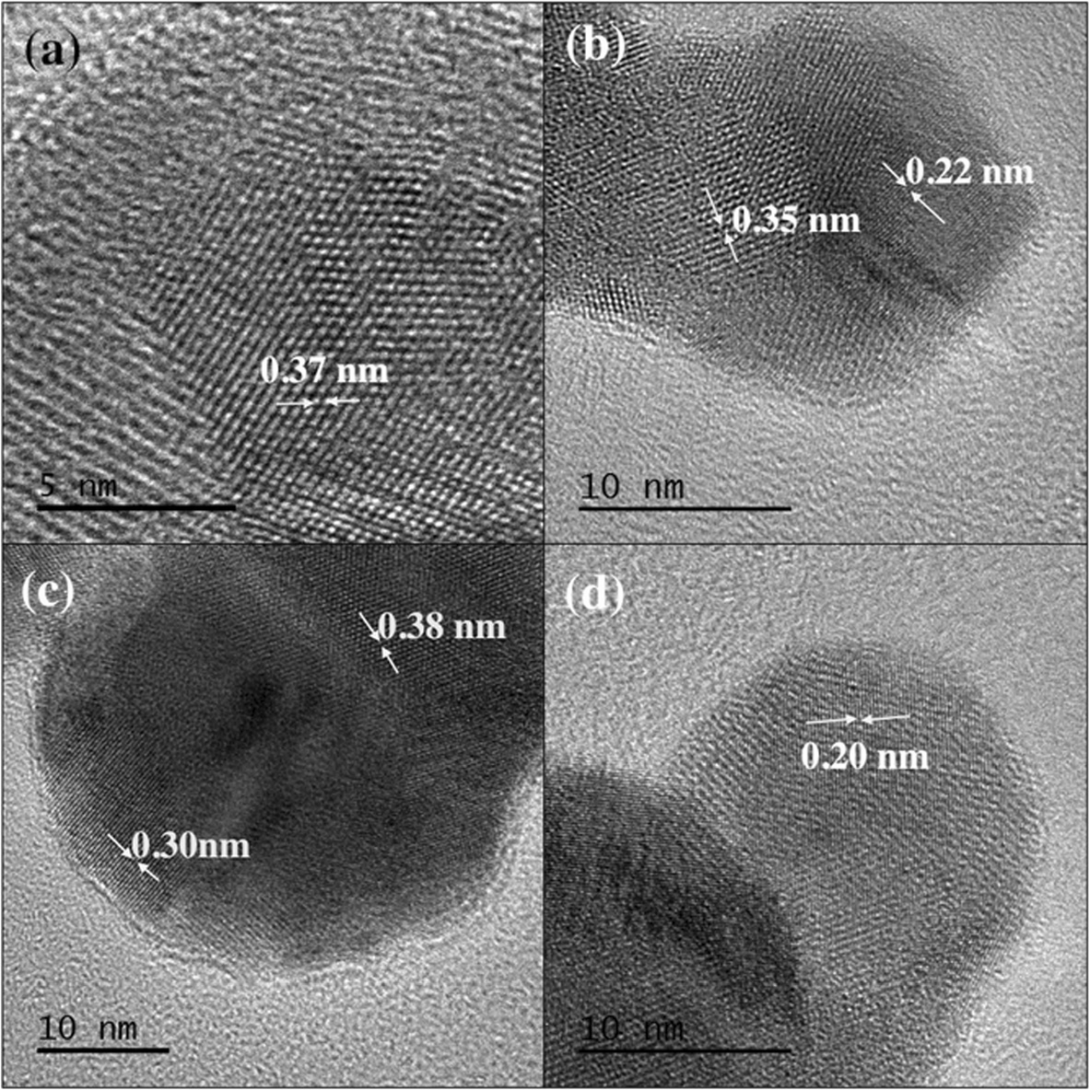
**a–d** HRTEM images of InSb with lattice spacing around 0.37 and 0.23 nm corresponding to the *h*, *k*, and *l* values of (111) and (220), respectively

**Fig. 5:**
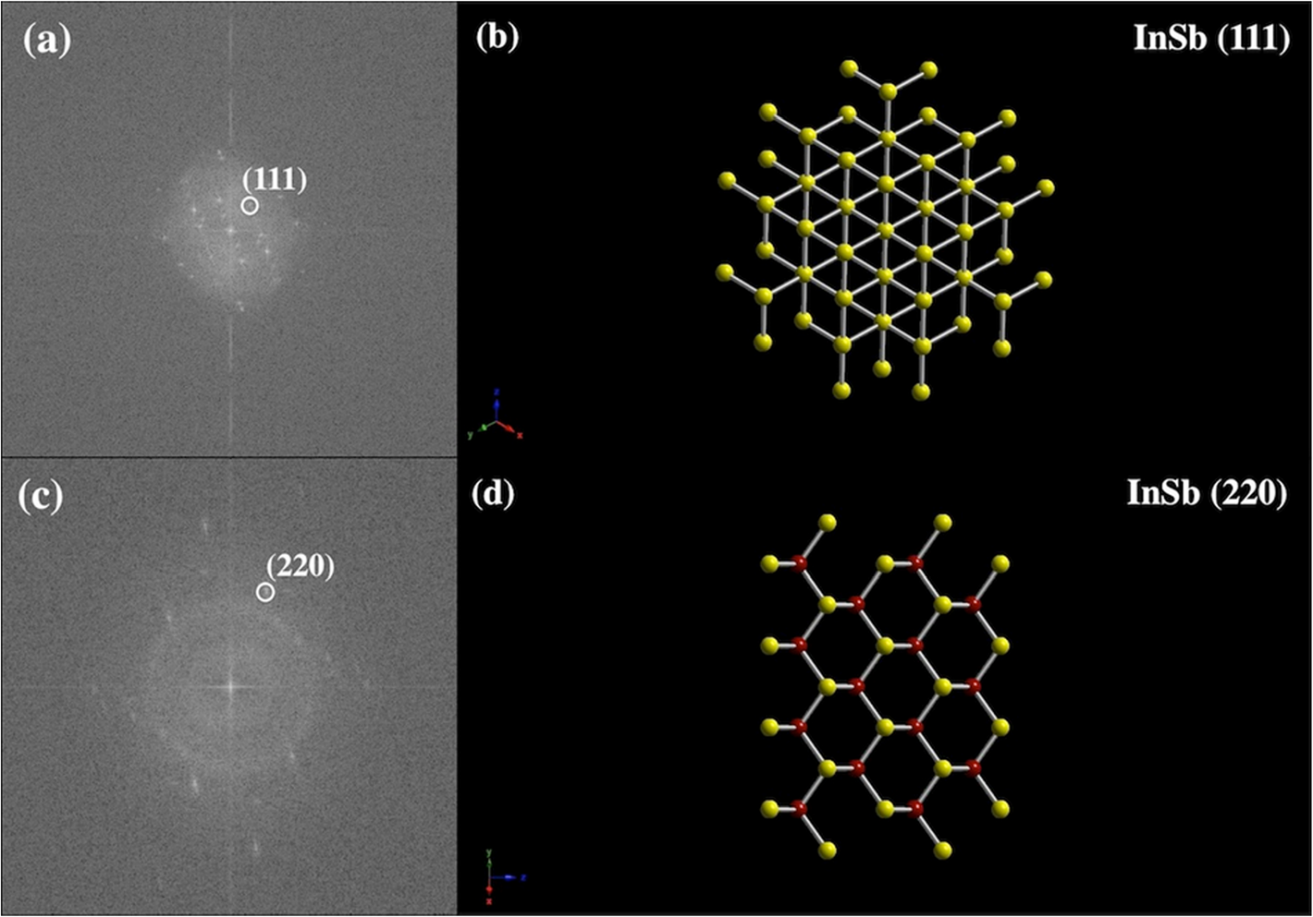
**a** FFT image of Fig. 4a indicating the dominant growth direction of (111) corresponding to the lattice spacing of 0.37 nm. **b** Model showing InSb crystal structure as seen from the (111) direction. **c** FFT image of Fig. 4d indicating the dominant growth direction of (220) corresponding to the lattice spacing of ~0.23 nm. **d** Model showing InSb crystal structure as seen from the (220) direction

**Fig. 6:**
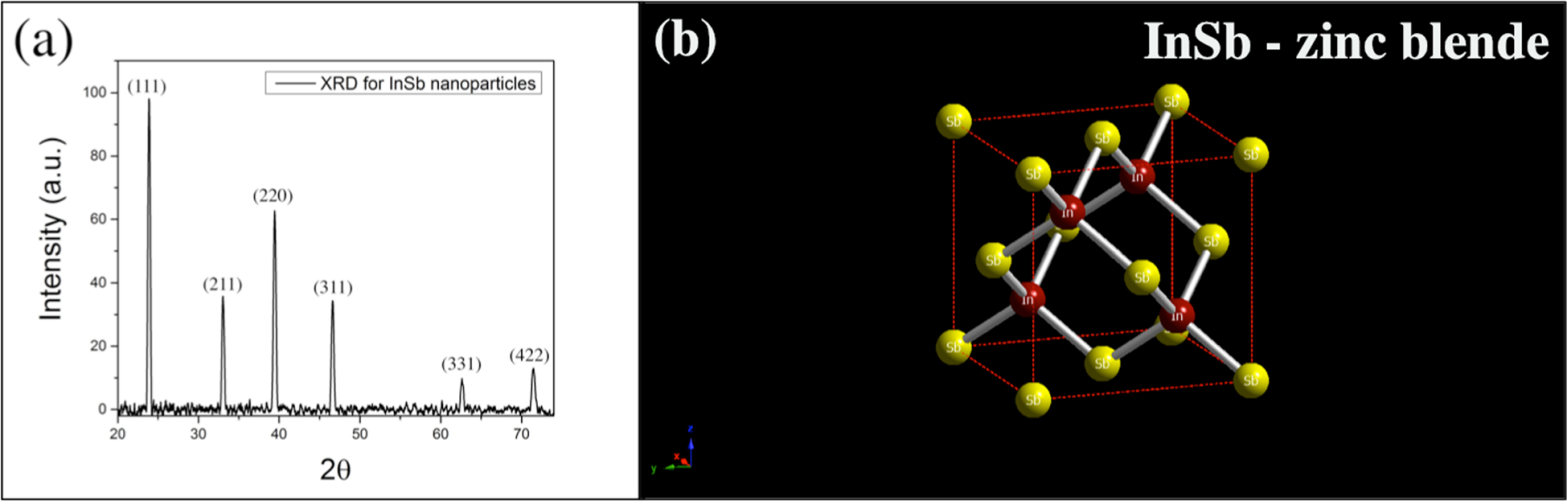
**a** XRD spectrum for as-deposited InSb NPs synthesized by IGC process. **b** Unit cell for zinc blende InSb

The structural characterization was carried out using HRTEM and XRD. Figure 4 shows various HRTEM images for InSb NPs deposited on lacey carbon TEM grid at room temperature. It can be seen from the HRTEM images that the particles are polycrystalline in nature. The two major lattice spacings observed are ~0.37 and ~0.23 nm. Fast Fourier transform (FFT) analysis was carried out for the HRTEM images seen in Fig. 4, and it was found that the lattice spacing of ~0.37 nm corresponds to the (111) direction and the lattice spacing of ~0.23 nm corresponds to the (220) direction. Figure 5a, c shows the FFT images for the HRTEM images in Fig. 4a, d, respectively. Figure 5b, d shows the model of InSb zinc blende structure as seen along (111) and (220) directions, respectively. As shown in Fig. 5b, along the (111) direction, one can only see the Sb (yellow) atoms and the separation in between these is around ~0.37 nm. This explains the lattice spacing of around 0.30, 0.35, 0.37, and 0.38 nm observed in the HRTEM images. Similarly, as shown in Fig. 5d, along the (220) direction, one can see both In (red) and Sb (yellow) atoms and the separation in between each neighboring red and yellow atoms is ~0.23 nm. This explains the lattice spacing of around 0.20 and 0.22 nm in the HRTEM images. The XRD spectrum obtained for InSb NPs can be seen in Fig. 6a. The NPs exhibited cubic symmetry with zinc blende structure (see Fig. 6b) corresponding to the space group of F-43m and with growth along the directions of (111), (211), (220), (311), (331), and (422). Amongst these, (111) and (220) directions were dominant. This confirms the observations obtained using HRTEM. The integral broadening *β* of the XRD peaks was measured for the XRD spectrum in Fig. 6a. Using *β*, the average grain size and the strain for the NPs were calculated using the Debye-Scherrer formula given by Eqs. 1 and 2.

1 $$ D=\frac{k\lambda }{\beta \cos \theta } $$

2 $$ \varepsilon =\frac{\beta }{4 \tan \theta } $$

Here, *D* is the crystallite size, *k* is the shape factor (~1), *λ* is the wavelength of the CuΚα radiations (~0.154 nm), *β* is the integral broadening of XRD peaks in radians, and *ε* is the lattice strain. The *β* parameter was corrected for instrumental broadening.

The mean value of the grain size was found to be 25.62 nm, and the value of strain in NPs was found to be 0.0015 nm. The value of *D* is comparable to the average particle size of NPs seen under the TEM.

**Fig. 7:**
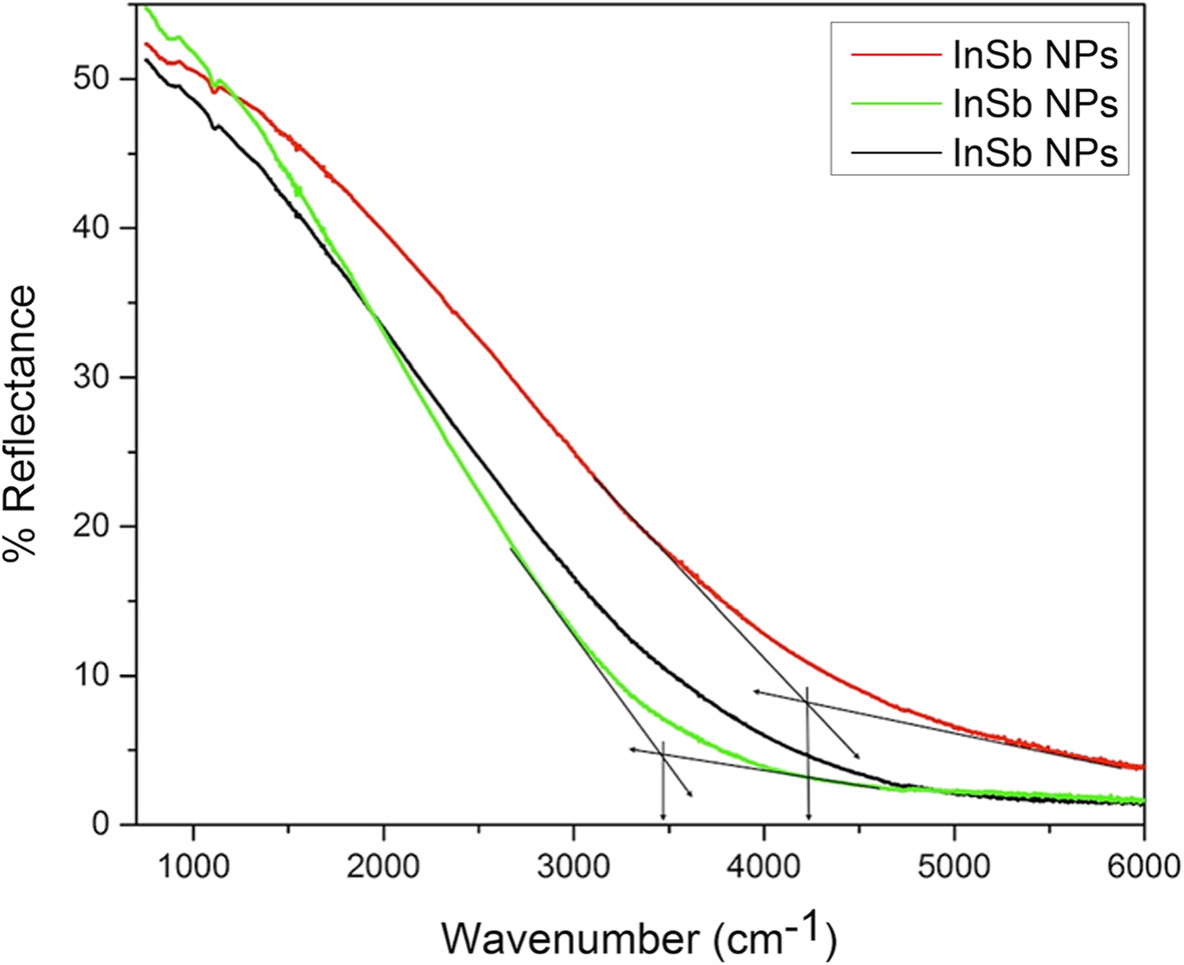
Reflectance spectrum for InSb NPs deposited on (111) p-type Si wafer using IGC process. The point of intersections indicated by *arrows* in the figure are 3470 and 4190 cm^−1^, which correspond to the energy of 0.43 and 0.52 eV. The different reflectance lines correspond to different areas of deposition for the same sample

**Fig. 8:**
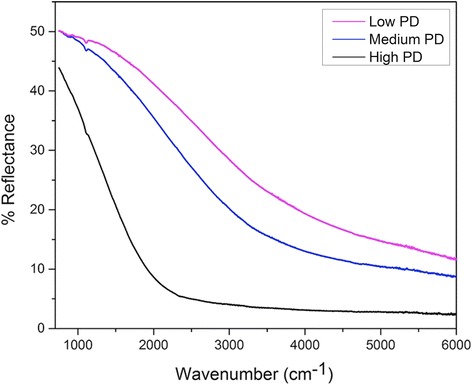
Decrease in the band gap of InSb NPs due to the increase in the particle density of NPs. The increased particle density causes aggregation in NPs and causes reduction in the quantum confinement effects

Finally, optical characterization of the NPs was obtained using FTIR. The NPs were deposited on Si wafers, and their reflectance was measured at room temperature, which was then used to calculate the band gap of the NPs. The reflectance spectrum obtained for the InSb NPs can be seen in Fig. 7. The point at which the reflectance goes to a minimum is the point where the band gap of the NPs is. After this point, the material starts to absorb the incident light and thus the reflectance approaches zero. The different reflectance lines in the figure correspond to different areas of deposition in the same sample. The arrows in the reflectance plot indicate the point of minimum reflectance, which is in the range of 3470–4190 cm^−1^. The energy corresponding to these wave numbers is around 0.43 and 0.52 eV, which is higher than the band gap of bulk InSb (~0.17 eV at 300 K). Thus, we observe a blue shift of around 0.26–0.35 eV due to the quantum confinement effects. The InSb NP size here is smaller than the exciton Bohr radius of InSb (~65 nm); thus, we observe an increase in the band gap of these NPs confirming quantum confinement effects in nanostructures. Figure 8 shows the reflectance spectrum for InSb NPs with different PDs. As we can see, there is a reduction in the band gap with increase in the PD. There are various factors that affect the absorbance and reflectance properties of NPs, namely, size, shape, and distance between the particles in the layers. For high-density samples, the distance between the particles is reduced and the NPs overlap. This also results into light being trapped in between the layers or overlapping NPs. Thus, the reflectance is relatively lower for samples with high density of particles. With further increase in the PD, it would start behaving as a thin film of InSb and the band gap would reduce further.

These NPs will be further characterized for photo detector applications, the results of which will be reported later.

## Conclusions

To summarize, we have demonstrated a vapor phase technique to synthesize InSb NPs useful for various electronics applications. The TEM studies showed bimodal distribution with average particle sizes of 13.70 and 33.20 nm. Structural characterization using HRTEM indicated that the NPs are polycrystalline in nature with a cubic symmetry and are formed of smaller crystallites. X-ray diffraction result indicates that the sample has a crystalline zinc blende structure. The average crystallite size of the NPs obtained using XRD peak broadening results and the Debye-Scherrer formula was 25.62 nm, and the value of strain in NPs was found to be 0.0015. The crystallite size of the NPs calculated using Debye-Scherrer’s formula is in agreement with the NP size obtained using TEM. Raman spectra shows a peak at 184.27 cm^−1^ corresponding to the LO mode of InSb, and EDX study shows that the NPs have 1:1 In-to-Sb ratio. This confirms the formation of single phase InSb NPs. The band gap of the NPs as calculated using reflectance mode of FTIR was in the range of 0.43–0.52 eV. Thus, a blue shift of 0.26–0.35 eV was observed in the band gap of these NPs showing the effects of quantum confinement in nanostructures. Increased particle density shows reduction in the quantum confinement effects causing decrease in the band gap of the InSb NPs.
